# Artemisinin Confers Neuroprotection against 6-OHDA-Induced Neuronal Injury In Vitro and In Vivo through Activation of the ERK1/2 Pathway

**DOI:** 10.3390/molecules28145527

**Published:** 2023-07-20

**Authors:** Qin Li, Shuai Li, Jiankang Fang, Chao Yang, Xia Zhao, Qing Wang, Wenshu Zhou, Wenhua Zheng

**Affiliations:** 1 Center of Reproduction, Development & Aging and Institute of Translation Medicine, Faculty of Health Sciences, University of Macau, Room 3057, Building E12, Taipa, Macau SAR 999078, China; liqin@zjams.com.cn (Q.L.); lishuai01090722@163.com (S.L.); yb57646@um.edu.mo (J.F.);; 2 School of pharmacy, Hangzhou Medical College, Hangzhou 310059, China; 3 Guangdong Provincial Engineering Research Center of Molecular Imaging, The Fifth Affiliated Hospital, Sun Yat-sen University, Zhuhai 519000, China; 4 Department of Neurology, Zhujiang Hospital of Southern Medical University, Guangzhou 510280, China; wqdennis@hotmail.com

**Keywords:** artemisinin, 6-OHDA, MPTP/MPP^+^, apoptosis, Parkinson’s disease

## Abstract

Parkinson’s disease (PD) is an age-related, progressive neurodegenerative disease characterized by the gradual and massive loss of dopaminergic neurons in the substantia nigra pars compacta (SNc). We have recently reported that artemisinin, an FDA-approved first-line antimalarial drug, possesses a neuroprotective effect. However, the effects and underlying mechanisms of artemisinin on Parkinson’s disease remain to be elucidated. In this study, we investigated the neuroprotective effects of artemisinin on 6-OHDA and MPP^+^ in neuronal cells and animal models, as well as the underlying mechanisms. Our results showed that artemisinin significantly attenuated the loss of cell viability, LDH release, elevated levels of reactive oxygen species (ROS), the collapse of the mitochondria trans-membrane potential and cell apoptosis in PC12 cells. Western blot results showed that artemisinin stimulated the phosphorylation of ERK1/2, its upstream signaling proteins c-Raf and MEK and its downstream target CREB in PC12 cells in a time- and concentration-dependent manner. In addition, the protective effect of artemisinin was significantly reduced when the ERK pathway was blocked using the ERK pathway inhibitor PD98059 or when the expression of ERK was knocked down using sgRNA. These results indicate the essential role of ERK in the protective effect of artemisinin. Similar results were obtained in SH-SY5Y cells and primary cultured neurons treated with 6-OHDA, as well as in cellular models of MPP^+^ injury. More interestingly, artemisinin attenuated PD-like behavior deficit in mice injected with 6-OHDA evaluated by behavioral tests including swimming test, pole-test, open field exploration and rotarod tests. Moreover, artemisinin also stimulated the phosphorylation of ERK1/2, inhibited apoptosis, and rescued dopaminergic neurons in SNc of these animals. Application of ERK pathway inhibitor PD98059 blocked the protective effect of artemisinin in mice during testing. Taking these results together, it was indicated that artemisinin preserves neuroprotective effects against 6-OHDA and MPP^+^ induced injury both in vitro and in vivo by the stimulation of the ERK1/2 signaling pathway. Our findings support the potential therapeutic effect of artemisinin in the prevention and treatment of Parkinson’s disease.

## 1. Introduction

Parkinson’s disease (PD) is an age-related progressive neurodegenerative disease and the second most frequent neurodegenerative disease after Alzheimer’s disease. The primary pathology of PD is characterized by the gradual and extensive loss of dopaminergic neurons in the substantia nigra pars compacta (SNc), leading to a significant decrease in dopamine (DA) levels [1]. The etiology of PD is currently unknown, but there was evidence suggesting the involvement of both genetic and environmental factors. Aging is considered the biggest risk factor for this disease [2]. Moreover, biochemical factors such as oxidative stress, neuroinflammation and mitochondrial toxins play important roles in the pathogenesis of PD [3,4]. Although current treatments help to alleviate the clinical symptoms, an effective drug that stops or prevents the progression of PD has yet to be developed.

The mechanisms underlying PD pathogenesis are still unclear, but it is widely accepted that oxidative stress, induced by the overproduction of reactive oxygen species (ROS) is a key pathogenic factor [5,6]. ROS causes oxidative damage to proteins, lipids, and DNA. It can also cause mitochondrial dysfunction and activate apoptotic signaling pathways, leading to nerve cell dysfunction and apoptosis, which subsequently leads to neurodegeneration. Therefore, anti-oxidative stress approaches have become important strategies for neuroprotection, as well as the prevention and treatment of PD [7].

6-hydroxydopamine (6-OHDA), MPTP (1-methyl-4-phenyl-1,2,3,6-tetrahydropyridine) and MPP^+^ (1-methyl-4-phenylpyridinium ion) are dopaminergic neurotoxic chemicals widely used to create cellular and animal models of PD. 6-OHDA is a toxic dopamine metabolite that undergoes rapid and nonenzymatic oxidation by molecular oxygen to form p-quinone and hydrogen peroxide [8]. This process damages dopaminergic neurons and contributes to the pathogenesis of PD. Additionally, 6-OHDA is taken up by the noradrenaline transporter and causes damage to noradrenergic neurons. MPTP or MPP^+^ is selectively taken up by the dopamine transporter and accumulates in dopaminergic neurons. moreover, they are well-known mitochondrial dopaminergic neurotoxins that trigger mitochondrial dysfunction and impair autophagy. The induction of ROS formation and oxidative stress are major mechanisms implicated in the neurotoxicity of 6-OHDA, MPTP and MPP^+^.

Artemisinin (ART) is originally extracted from the Chinese medicinal plant known as Artemisia annua (qinghao). It is a highly effective anti-malarial drug that has been used for decades in clinics for the treatment of malaria. Artemisinin can pass through the blood-brain barrier (BBB) without any noticeable side effects. In addition, artemisinin has been shown to have antioxidant, anti-inflammatory, anti-viral, and anti-bacterial effects. Most importantly, we have recently demonstrated that artemisinin exhibits significant neuroprotective effects, particularly in cellular and animal models of age-related macular degeneration and Alzheimer’s disease [9,10,11]. However, the effect and underlying mechanism of artemisinin on PD is not known at present.

In the present study, we investigated the neuroprotective effects of artemisinin in both in vitro and in vivo models of PD, as well as its potential molecular mechanisms. Our results showed that artemisinin was able to protect neuronal cells against the toxicity of 6-hydroxydopamine (6-OHDA) and MPTP/MPP^+^ both in vitro and in vivo by stimulating the activation of the ERK pathway. These results support the potential application of artemisinin in the prevention and treatment of PD.

## 2. Results

### 2.1. Artemisinin Attenuated the Decrease in Cell Viability and Cell Cytotoxicity Caused by 6-OHDA in PC12 Cells

**Figure 1:**
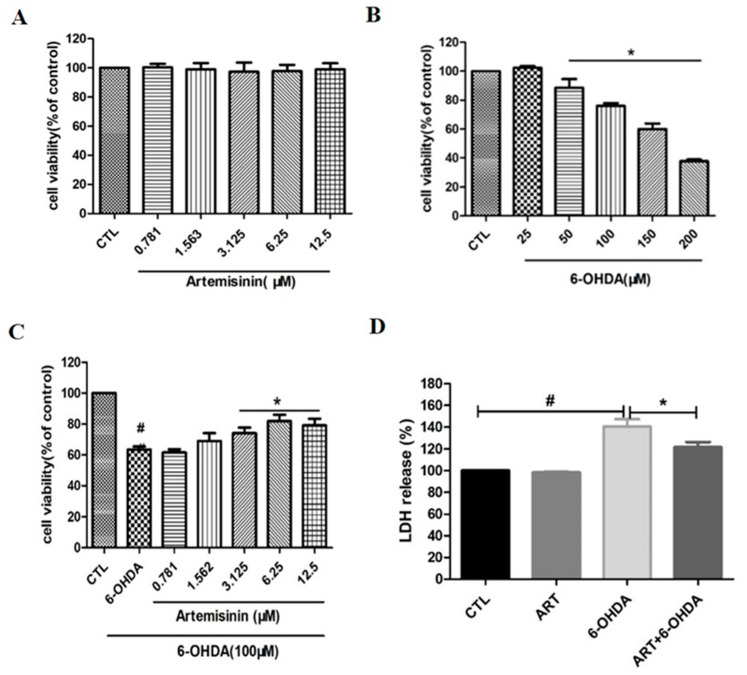
Artemisinin attenuated the decrease in cell viability caused by 6-OHDA in PC12 cells. (**A**) The cytotoxicity of artemisinin. Cells were treated with different concentrations of artemisinin for 24 h and the cell viability was measured by MTT assay. (**B**) The cytotoxicity of 6-OHDA. Cells were treated with different concentrations of 6-OHDA for 24 h. The cell viability was measured by MTT assay. (**C**,**D**) Cells were pretreated with artemisinin at different concentrations for 2 h and then incubated with or without 100 μM 6-OHDA for a further 24 h. The cell viability and cell cytotoxicity were measured by MTT (**C**) and LDH (**D**) assays. Data representing mean ± SD, * *p* < 0.05, # *p* < 0.05 were considered significantly different.

To evaluate the toxic effect of artemisinin and 6-OHDA, PC12 cells were treated with different concentrations of artemisinin (ranging between 0–12.5 μM) or 6-OHDA (ranging between 0–200 μM) for 24 h and the viability of cells was evaluated by MTT assay. As shown in Figure 1A, artemisinin did not have any obvious cytotoxic effect in PC12 cells ranging from 0 to 12.5 μM while 6-OHDA concentration-dependently induced a reduction of cell viability. The toxicity of 6-OHDA was significant at 100 μM (cell viability decreases about 30–40%) and this concentration was chosen for the following experiments (Figure 1B). To evaluate the protective effect of artemisinin against 6-OHDA induced cell death, PC12 cells were pre-treated with artemisinin for 2 h before being exposed to 100 μM 6-OHDA for 24 h. As shown in Figure 1C, artemisinin significantly attenuated the cell viability loss induced by 6-OHDA. Further evaluation of the effect of artemisinin and 6-OHDA on LDH release showed that 100 μM 6-OHDA induced a significant increase in the LDH released into the medium compared with control cells while artemisinin pre-treatment (6.25 μM) reduced the LDH release induced by 6-OHDA (Figure 1D).

### 2.2. Artemisinin Inhibited 6-OHDA-Induced ROS Accumulation, Loss of the Mitochondria Membrane Potential and Apoptosis in PC12 Cells

**Figure 2:**
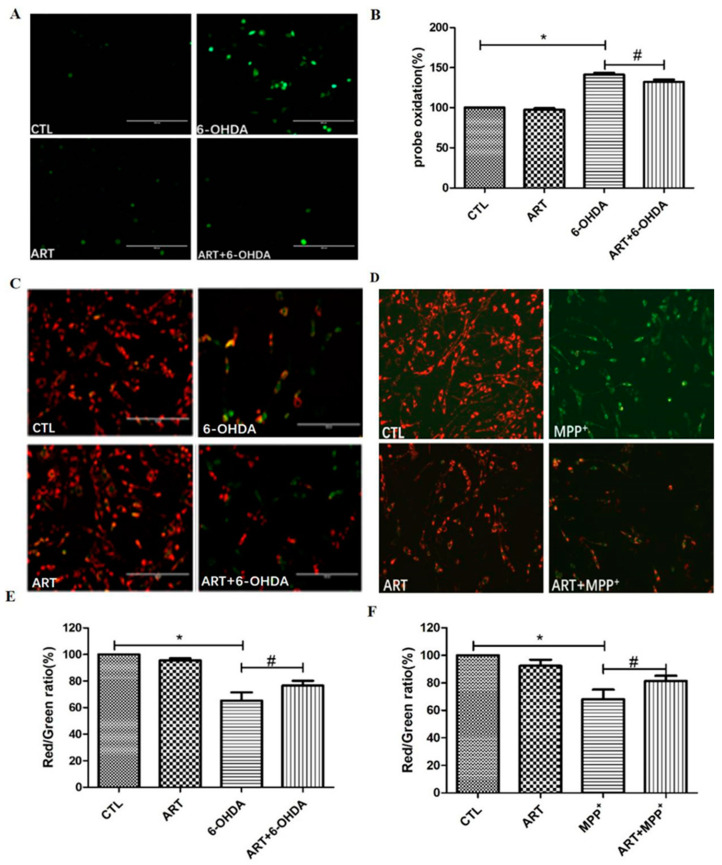
Artemisinin inhibited 6-OHDA-induced elevated ROS levels and 6-OHDA and MPP^+^-induced mitochondrial membrane potential loss in PC12 cells. Cells were pretreated with 6.25 μM Artemisinin and then incubated with or without 100 μM 6-OHDA for further 24 h. (**A**) The intracellular ROS was measured by DCFH-DA reagent. Micrographs of fluorescent labeled cells stained by DCFH-DA reagent for ROS (scale bars, 200 μm). (**B**) Quantitative analysis of A. Cells were pretreated with 6.25 μM Artemisinin and then incubated with or without 100 μM 6-OHDA or 1000 μM MPP^+^ for further 24 h. (**C**,**D**) The decline of the mitochondrial membrane potential was reflected by the shift of fluorescence from red to green indicated by JC-1 (scale bars, 100 μm). (**E**,**F**) Quantitative analysis of (**C**,**D**). Data represent mean ± SD, * *p* < 0.05, # *p* < 0.05 were considered significantly different.

**Figure 3:**
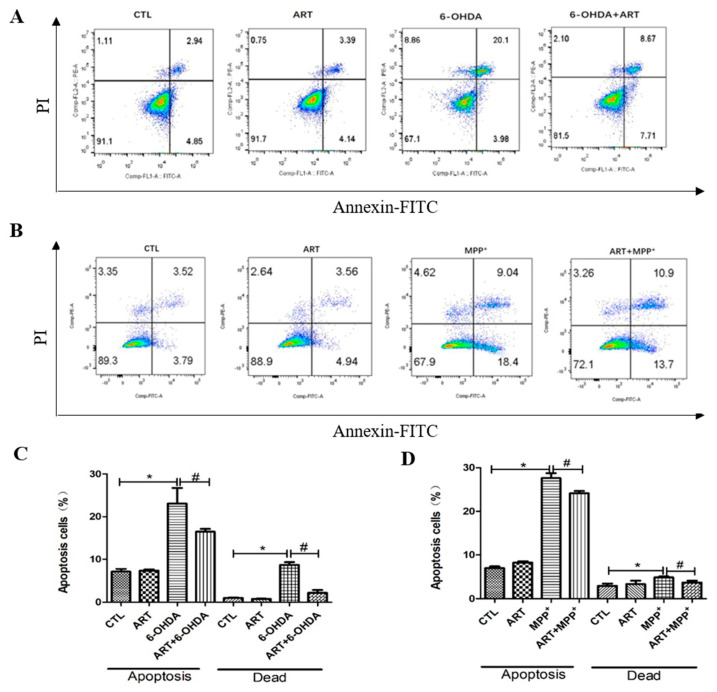
Artemisinin suppressed 6-OHDA and MPP^+^-induced apoptosis in PC12 cells. Cells were pre-treated with 6.25 μM artemisinin for 2 h and then induced with or without 100 μM 6-OHDA or 1000 μM MPP^+^ for another 24 h (**A**,**B**) Photographs of the representative cultures measured by flow cytometry. (**C**,**D**) Quantitative analysis of A and B. Data represent mean ± SD, * *p* < 0.05, # *p* < 0.05 were considered significantly different.

The evaluation of the effect of artemisinin and 6-OHDA on intracellular ROS accumulation, Results showed that 6.25 μM artemisinin pre-treatment significantly reversed the 100 μM 6-OHDA induced the increase of intracellular ROS (Figure 2A,B). In addition, artemisinin pre-treatment (6.25 μM) was also able to reverse the loss of mitochondrial membrane potential induced by the treatment of cells with 100 μM 6-OHDA or 1000 μM MPP^+^ for 24 h (Figure 2C–F). Further investigation of the neuroprotective effect of artemisinin upon 6-OHDA or MPP^+^-induced apoptosis by FACS analyses of Annexin-V-FITC labeled cells, it revealed that artemisinin pre-treatment (6.25 μM) also significantly reversed the 6-OHDA or MPP^+^-induced the increase of cell apoptosis (Figure 3).

### 2.3. Artemisinin Increased the Phosphorylation/Activation of ERK1/2, and ERK’s Upstream Proteins Raf and MEK, and ERK’s Downstream Protein CREB in a Concentration- and Time-Dependent Manner in PC12 Cells

**Figure 4:**
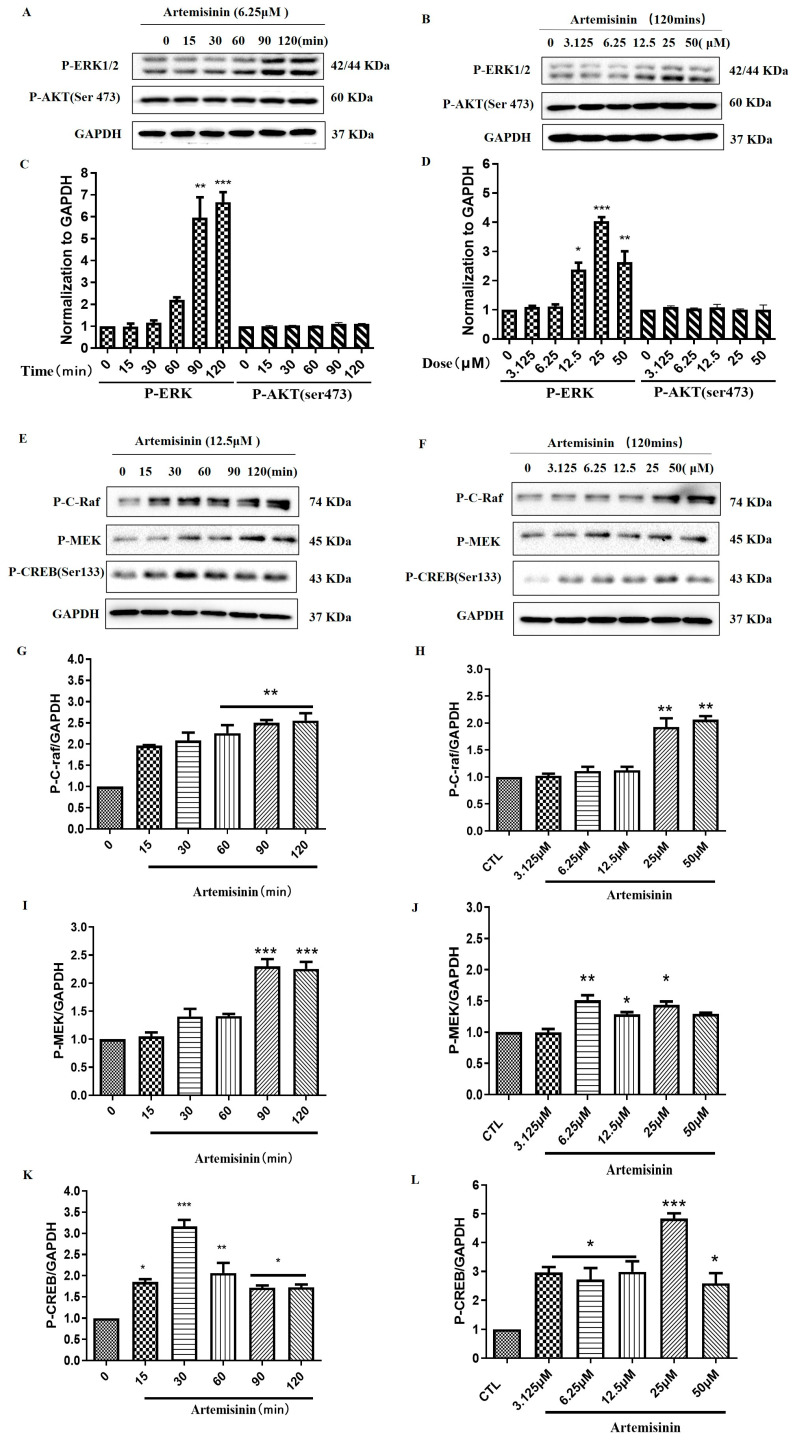
Artemisinin stimulated the phosphorylation/activation of ERK1/2, and C-Raf, MEK and CREB at a concentration- and time-dependent manner in PC12 cells. (**A**,**B**) The PC12 cells were collected with artemisinin treatment for different times (0, 15, 30, 60, 90 and 120 min) at 6.25 μM, and at different concentrations (3.125, 6.25, 12.5, 25 and 50 μM) for 120 min. The phosphorylation of ERK1/2, AKT and expression of GAPDH were detected by western blot. (**C**,**D**) Quantitative analysis of A and B. (**E**,**F**) The PC12 cells were collected with artemisinin treatment for different times (0, 15, 30, 60, 90 and 120 min) at 12.5 μM, and at different concentrations (3.125, 6.25, 12.5, 25 and 50 μM) for 120 min. The expression of P-C-Raf, P-MEK, P-CREB and GAPDH were detected by western blot. (**G**–**L**) Quantitative analysis of (**G**,**H**). Data represent mean ± SEM, * *p* < 0.05, ** *p* < 0.01, *** *p* < 0.001 were considered significantly different.

After the confirmation of the neuroprotective role of artemisinin against 6-OHDA and MPP^+^-induced injury, we aimed to investigate the molecular mechanisms underlying artemisinin action, by measuring the phosphorylated protein levels of ERK1/2 and AKT. As demonstrated in Figure 4A–D, artemisinin time- and concentration-dependently stimulated the phosphorylation of ERK1/2, while having no effect on AKT phosphorylation, indicating the involvement of the ERK pathway. Then, we studied the effect of artemisinin on upstream and downstream signaling proteins of ERK1/2 cascade such as C-Raf and MEK and CREB. Figure 4E–L showed that artemisinin also stimulated the phosphorylation of C-Raf, MEK and CREB in a time- and dose-dependent manner. These findings suggest that artemisinin may confer its protective effect by regulating the Raf-MEK-ERK-CREB signaling pathway.

### 2.4. The ERK1/2 Pathway Inhibitor PD98059 or Silencing of ERK1/2 Blocked the Protective Effect of Artemisinin in PC12 Cells

**Figure 5:**
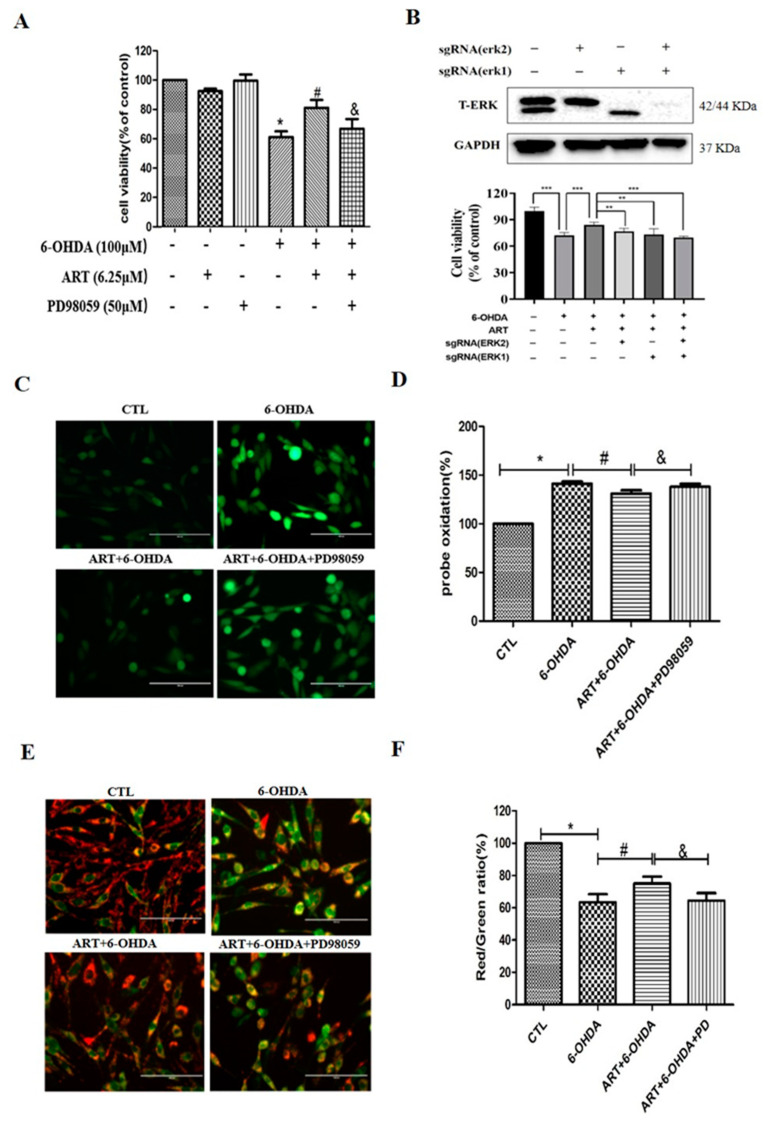
ERK1/2 specific inhibitor blocked the protective effect of artemisinin in PC12 cells. (**A**) Cells were pre-treated with 50 µM PD98059 (ERK inhibitor) for 60 min, treated with 6.25 µM artemisinin for 2 h and then incubated with or without 100 µM 6-OHDA for another 24 h. The cell viability was determined by MTT assay. (**B**) Cells were transfected with ERK1/2 sgRNA, pre-treated with 6.25 μM artemisinin for 2 h and then incubated with or without 100 μM 6-OHDA for another 24 h. Cell viability was measured by MTT assay. (**C**) Effect of PD98059 on artemisinin protective action against 6-OHDA-induced increase of intracellular ROS levels. Micrographs of fluorescent labeled cells stained by DCFH-DA reagent for ROS (scale bars, 100 μm). (**D**) Quantification of (**C**). (**E**) Effect of PD98059 on artemisinin protective action against 6-OHDA-induced decline of the mitochondrial membrane potential. The decline of the mitochondrial membrane potential was reflected by the shift of fluorescence from red to green indicated by JC-1 (scale bars, 100 μm). (**F**) Quantification of (**E**). Data represent mean ± SD, * *p* < 0.05, ** *p* < 0.01, *** *p* < 0.001, & *p* < 0.05, # *p* < 0.05 were considered significantly different.

**Figure 6:**
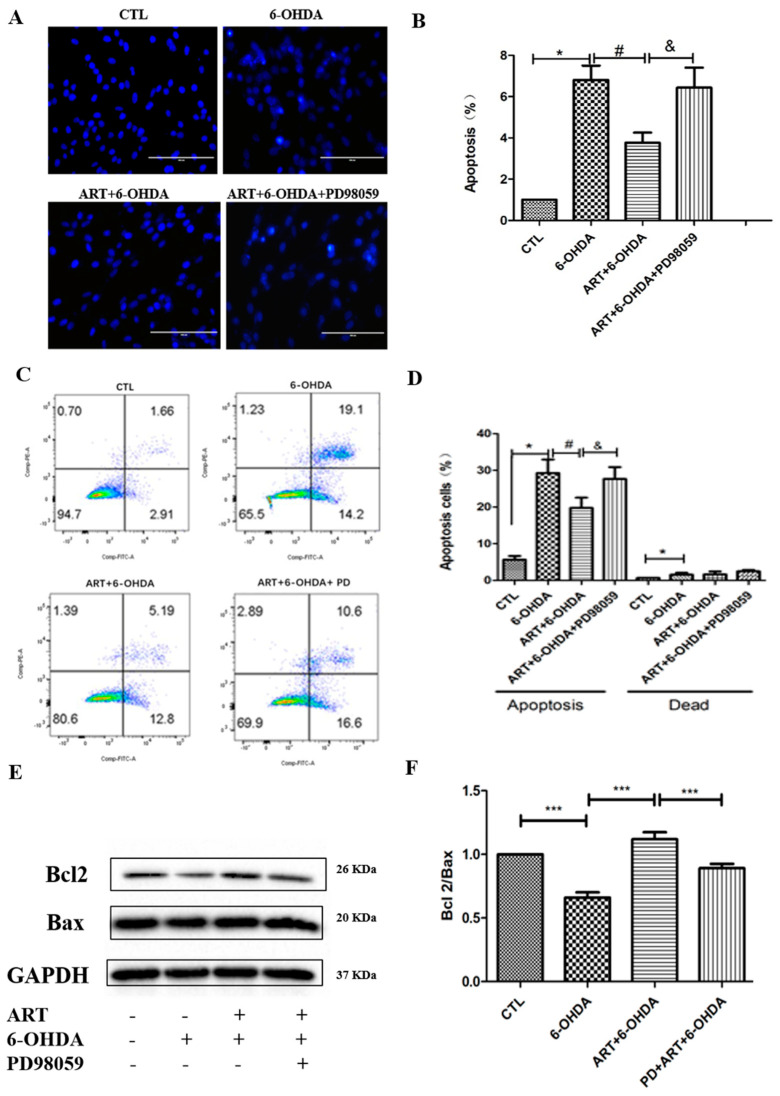
ERK1/2 specific inhibitor blocked the anti-apoptotic effect of artemisinin in PC12 cells. (**A**) Hoechst staining was used to evaluate the effect of PD98059 on artemisinin action against 6-OHDA-induced cell apoptosis. Micrographs of fluorescent labeled cells by Hoechst staining (scale bars, 200 μm). (**B**) Quantification of (**A**). (**C**) Photographs of representative cultures measured by flow cytometry. (**D**) Quantification of (**C**). (**E**) The expression of Bcl2, Bax and GAPDH were detected by western blot. (**F**) Quantification of Bcl2/Bax ratio. Data represent mean ± SD, * *p* < 0.05, *** *p* < 0.001, & *p* < 0.05, # *p* < 0.05 were considered significantly different.

To further elucidate the role of ERK1/2 in the protective effect of artemisinin against 6-OHDA-induced cell injury, we tested the effect of artemisinin in PC12 cells when ERK1/2 expression was inhibited by ERK1/2 inhibitor PD98059 or sgERK1 and sgERK2. (Figure 5). Obtained results from the MTT assay demonstrated that PD98059 inhibited the protective effect of artemisinin against 6-OHDA-induced cell viability loss (Figure 5A). Moreover, western blot analysis and MTT assay results showed that the blockage of the expression of ERK1/2 by its specific sgRNA attenuated the protective effect of artemisinin against 6-OHDA–induced injury (Figure 5B). The beneficial effect of artemisinin (6.25 μM) on intracellular ROS levels and mitochondrial membrane potential (Δψm) was also blocked upon inhibition of ERK1/2 with PD98059 (Figure 5C–F). Similarly, PD98059 blocked the anti-apoptotic effect of artemisinin as indicated by Hochest DNA staining nuclei morphological changes (Figure 6A,B) and FACS analyses of Annexin-V-FITC labeling (Figure 6C,D). Furthermore, artemisinin increased 6-OHDA-induced decrease of Bcl2/Bax expression ratio was also prevented by PD98059 (Figure 6E,F). Taken together, these data suggest that artemisinin confers protection against 6-OHDA-induced cell damage by stimulating the ERK1/2 pathway.

### 2.5. Artemisinin Protected SH-SY5Y Cells and Primary Cultured Neuronal Cells against 6-OHDA and MPP^+^ Induced Cell Viability Loss

**Figure 7:**
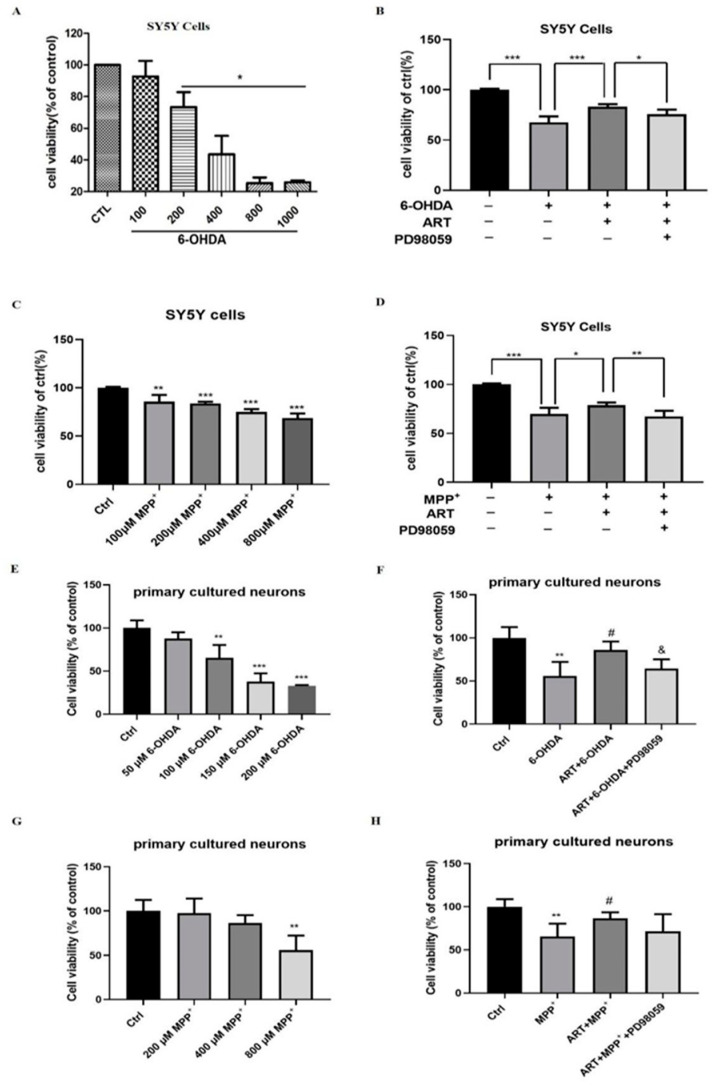
Artemisinin attenuated the decrease in cell viability caused by 6-OHDA and MPP^+^ in SH-SY5Y cells and primary cultured neurons. (**A**) The cytotoxicity of 6-OHDA in SH-SY5Y cells. Cells were treated with different concentrations of 6-OHDA for 24 h and cell viability was measured using the MTT assay. (**B**) Cells were pre-treated with 25 µM PD98059 (ERK inhibitor) for 40 min, treated with 6.25 µM artemisinin for 2 h and then incubated with or without 6-OHDA for another 24 h. The cells viability was determined by MTT assay. (**C**) The cytotoxicity of MPP^+^ in SH-SY5Y cells. Cells were treated with different concentrations of MPP^+^ for 24 h and cell viability was measured using the MTT assay. (**D**) Cells were pre-treated with 25 µM PD98059 (ERK inhibitor) for 40 min, treated with 6.25 µM artemisinin for 2 h and then incubated with or without MPP^+^ for another 24 h. The cells viability was determined by MTT assay. (**E**) The cytotoxicity of 6-OHDA in primary cultured neurons. Cells were treated with different concentrations of 6-OHDA for 24 h and cell viability was measured using the MTT assay. (**F**) Primary cultured neurons were pre-treated with 20 µM PD98059 (ERK inhibitor) for 40 min, treated with 6.25 µM artemisinin for 2 h and then incubated with or without 6-OHDA for another 24 h. The cells viability was determined by MTT assay. (**G**) The cytotoxicity of MPP^+^ in primary cultured neurons. Cells were treated with different concentrations of MPP^+^ for 24 h and cell viability was measured using the MTT assay. (**H**) Cells were pre-treated with 20 µM PD98059 (ERK inhibitor) for 40 min, treated with 6.25 µM artemisinin for 2 h and then incubated with or without MPP^+^ for another 24 h. The cells viability was measured using the MTT assay. Data represent means ± SD, * *p* < 0.05, ** *p* < 0.01, *** *p* < 0.001, & *p* < 0.05, # *p* < 0.05 were considered significantly different.

The possible neuroprotective effect of artemisinin against 6-OHDA and MPP^+^-in-duced cell injury in SH-SY5Y cells and primary cultured neurons was also assessed. MTT results revealed that MPP^+^ or 6-OHDA caused a concentration-dependent toxicity in SH-SY5Y cells and primary cultured neurons (Figure 7A,C,E,F), while artemisinin pre-treatment protected the cells from MPP^+^ or 6-OHDA injury. In addition, the protective effect of artemisinin was also blocked upon inhibition of ERK1/2 with PD98059 (Figure 7B,D,F,H). These results are similar to the ones obtained with PC12 cells model.

### 2.6. Artemisinin Attenuated the Behavioral Deficits in PD Mice Models Induced by 6-OHDA and MPTP

**Figure 8:**
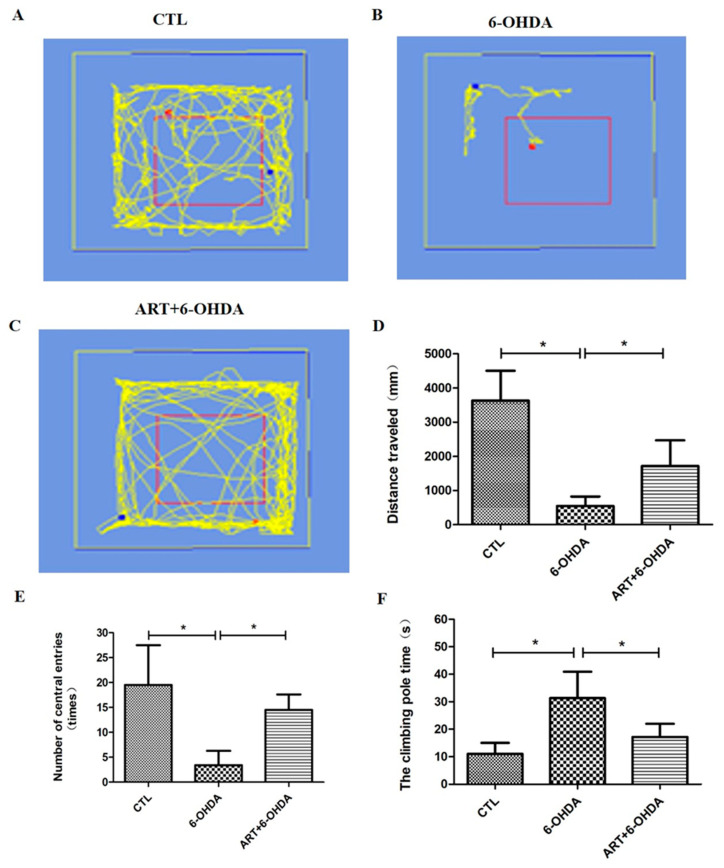
Artemisinin attenuated the behavioral deficits of 6-OHDA-induced PD mice model observed in open field and pole tests. (**A**–**C**) Representative pictures of the open field exploration test in each group. (**D**) The distance traveled in the open field exploration test. (**E**) Number of central entries in the open field exploration test. (**F**) The climbing time of each group in the pole-test. Data represent means ± SD, * *p* < 0.05 were considered significantly different.

**Figure 9:**
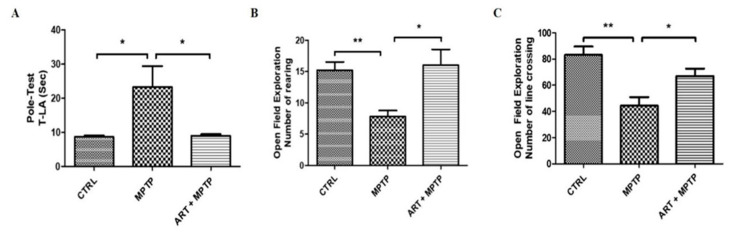
Artemisinin attenuated the behavioral deficits in the MPTP-induced PD mice model observed in the open field and pole tests. (**A**) The climbing time of each group in pole-test; (**B**) Number of rearing in the open field exploration test; (**C**) Number of line crossings in the open field exploration test. Data represent means ± SD, * *p* < 0.05, ** *p* < 0.01 were considered significantly different.

The behavioral assessment of PD mice showed that artemisinin treatment significantly attenuated the behavioral deficits in 6-OHDA-induced PD mice model observed in the open field exploration and pole tests. 6-OHDA mice exhibited a significant reduction of the traveled distance and the number of central entries in the open field test in comparison with the animals from the control group (Figure 8D,E). Artemisinin treatment significantly improved the traveled distance (Figure 8D) and the number of central entries (Figure 8E) in comparison with 6-OHDA model animals. The time spent to climbing the pole by 6-OHDA model animals was significantly longer compared with the animals from the control group, and artemisinin treatment significantly reduced the climbing pole time of 6-OHDA mice (Figure 8F). Similar results were obtained in MPTP-induced PD mice model (Figure 9) suggesting that artemisinin treatment significantly attenuated the behavioral deficits of both 6-OHDA and MPTP-induced PD mice models.

### 2.7. Artemisinin Treatment Stimulated the Phosphorylation of ERK1/2 in 6-OHDA-Induced PD Mice Model and This Effect Was Blocked by PD98059

**Figure 10:**
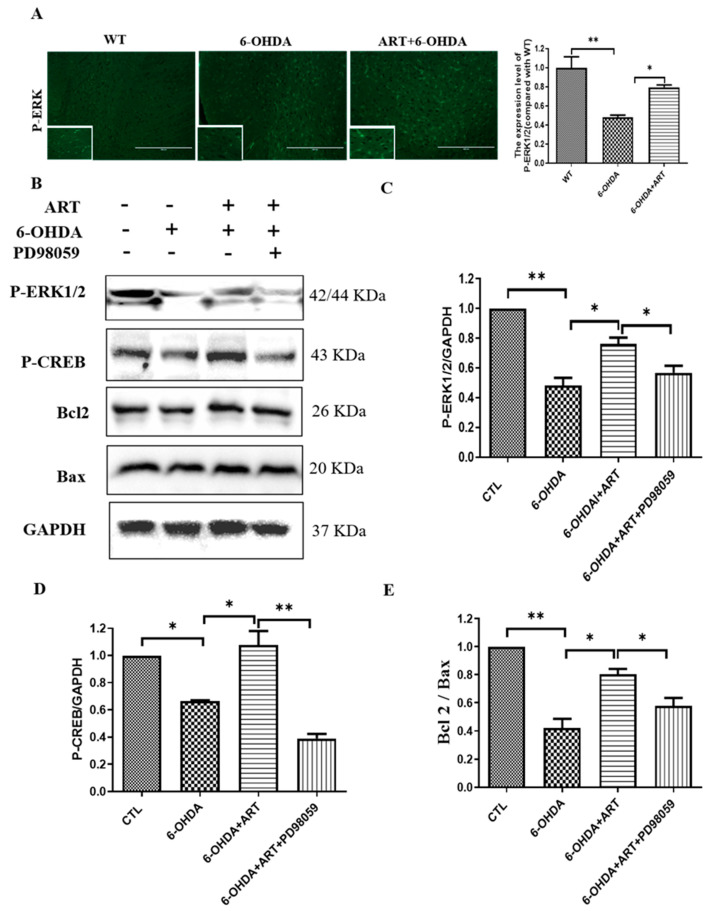
Artemisinin treatment activated the phosphorylation of ERK1/2 in 6-OHDA-induced PD mice model and pretreatment with PD98059 reversed this effect. (**A**) The expression of p-ERK1/2 in substantia nigra was detected by immunofluorescence and analyzed by Image pro plus software. (**B**,**C**) Effect of ERK inhibitor PD98059 pre-treatment on artemisinin neuroprotective action on 6-OHDA-induced PD mice model. (**B**) The expression levels of p-ERK, p-CREB, Bax, Bcl2 and GAPDH were detected by western blotting. (**C**–**E**) Quantification of the western blotting results. Data represent means ± SD, * *p* < 0.05, ** *p* < 0.01 were considered significantly different.

**Figure 11:**
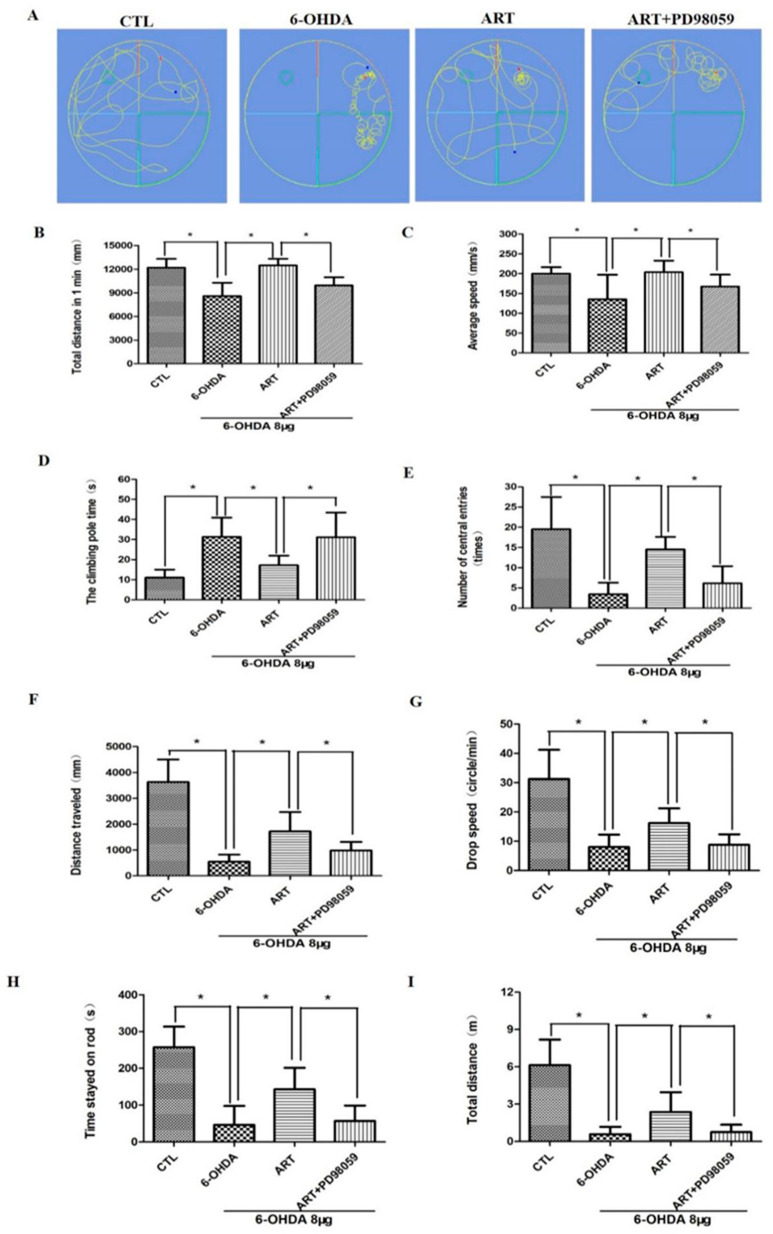
PD98059 pretreatment reversed the effect of artemisinin in the behavioral deficits. (**A**) Representative pictures of the swimming test in each group. (**B**) The distance traveled for 1 min of each group in the swimming test. (**C**) The average speed of each group in the swimming test. (**D**) The climbing time of each group in the pole-test. (**E**) The number of central entries of each group in the open field exploration test. (**F**) The distance traveled of each group in the open field exploration test. (**G**) The drop speed of each group in the rotarod test. (**H**) The time stayed on rod of each group in the in the rotarod test; (**I**) The total distance of each group in the rotarod test. Data represent means ± SD, * *p* < 0.05 were considered significantly different.

**Figure 12:**
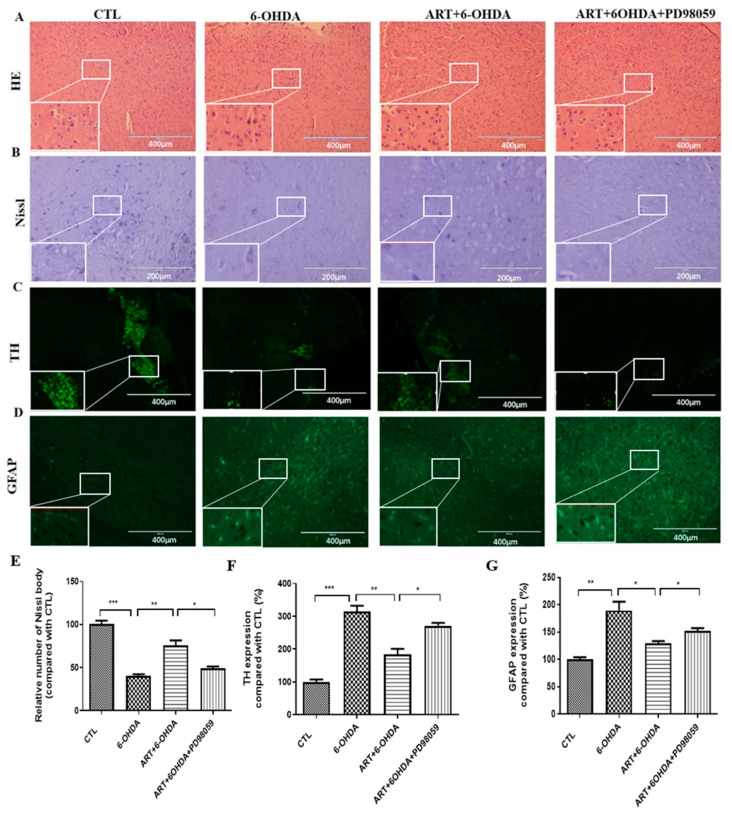
Artemisinin attenuated the pathological damage in 6-OHDA-induced PD mice model and these effects can be reversed by PD98059 treatment. (**A**) Representative images of H&E staining in substantia nigra of brain. Scale bars 200 μm (**B**) Representative images of Nissl staining in substantia nigra of brain, Scale bars 100 μm. (**C**) Representative images of TH immunofluorescence in substantia nigra of brain. (**D**) Representative images of GFAP immunofluorescence in substantia nigra of brain. Scale bars 200 μm. (**E**) Quantification of Nissl staining; (**F**) Quantification of TH immunofluorescence; (**G**) Quantification of GFAP immunofluorescence. Data represent means ± SD, * *p* < 0.05, ** *p* < 0.01 *** *p* < 0.001 were considered significantly different.

In this study, 6-OHDA treatment induced a downregulation of p-ERK1/2 expression levels. Upon artemisinin treatment, the phosphorylation levels of ERK1/2 significantly increased as denoted by immunofluorescence and western blot analysis (Figure 10). Pretreatment of the animals with the ERK1/2 inhibitor PD98059 prevented artemisinin-induced increase of the phosphorylation levels of ERK1/2 and of the apoptosis related markers Bax and Bcl2 (Figure 10B–D). Moreover, pretreatment of animals with the PD98059 inhibited the neuroprotective effect of artemisinin on the behavioral deficits (Figure 11) and brain pathological changes (Figure 12). As shown in Figure 11A–C, the traveled distance and the average speed of 6-OHDA animals in the swimming test, were significantly shorter in comparison with the animals from the control group. Artemisinin treatment significantly increased the traveled distance and the average speed of 6-OHDA model mice, and these effects were inhibited by PD98059. The beneficial effects of artemisinin in the time spent climbing the pole in the pole test and in the number of central entries and distance traveled in the open field test were also prevented by PD98059. Other behavioral parameters, including the drop speed, the time spent on the rod and the total distance in the rotarod were also significantly improved in 6-OHDA animals treated with artemisinin. Similarly, PD98059 pretreatment inhibited artemisinin protective action. Further assessment of the impact of artemisinin in the pathological changes of PD mice model’s brains revealed that artemisinin promoted a reduction of the neurodegenerative changes (Figure 12A) and the increase of the number of Nissl bodies of 6-OHDA mice (Figure 12B). Tyrosine hydroxylase (TH) is the rate-limiting enzyme for dopamine synthesis in the brain and a well-known, important primary factor directly involved in the pathogenesis of PD, which is mainly caused by dopaminergic neuronal loss in the substantia nigra. Therefore, we detected the expression levels of TH in the brain of each group animals. As shown in Figure 12C, 6-OHDA mice exhibited an obvious decrease of TH expression levels that was reversed by artemisinin treatment. 6-OHDA mice also exhibited an increased number of astrocyte-positive cells that was decreased by artemisinin (Figure 12D). Pretreatment with PD98059 reversed all these artemisinin effects.

## 3. Discussion

Parkinson’s disease (PD) is an age-related progressive neurodegenerative disease that involves a complex series of biochemical and molecular mechanisms. These mechanisms include oxidative stress, neuroinflammation, and mitochondrial dysfunction [7,12,13]. So far, effective therapies and new drugs that can prevent neuronal loss are still limited, highlighting the need for the development of innovative therapeutic approaches. As oxidative stress is one of the most significant factors contributing to injury in PD [6], recent studies have focused on exploring the potential use of antioxidants in the treatment of this condition. For example, nobiletin, a citrus flavonoid, was found to relieve oxidative stress, suggesting its potential use in the treatment and prevention of neurodegenerative diseases such as PD [14]. Additionally, it was recently reported that foods rich in antioxidants, such as vitamin C, vitamin E, carotenoids, selenium, and polyphenols may improve intracellular redox homeostasis to prevent or delay PD pathology [15]. Artemisinin has shown antioxidant capacities in various types of cells [11,16,17,18]. Several studies have shown that artemisinin provides neuroprotection by resisting oxidative stress induced by β-amyloid (Aβ), hydrogen peroxide (H_2_O_2_), sodium nitroprusside (SNP) and glutamate [9,10,19,20]. However, studies reporting on the potential of artemisinin against PD are scarce.

Recently, there has been a growing interest in the role of mitochondrial abnormalities in PD, and researchers have been investigating mitochondria as potential targets for therapeutic interventions [21]. For example, the antioxidant coenzyme Q, which functions in concert with certain mitochondrial enzymes, was reported to have the ability to reduce the worsening of symptoms associated with PD [22].

As obtaining and maintaining human dopaminergic neurons (which are primarily involved in PD) primary cells is challenging, current in vitro research on PD primarily focuses on establishing permanent neuronal models. PC12 cells from adrenal pheochromocytoma and SH-SY5Y cells from neuroblastoma share many characteristics with primary sympathetic neurons and are frequently utilized in the development of in vitro models for PD [23,24]. Some studies have also reported the use of PC12 cells and SH-SY5Y cells to establish a PD oxidative stress model. These studies have examined the effects of artemisinin in improving PD by inhibiting autophagy and exerting neuroprotective effects. They provide an important reference for establishing an in vitro model of PD [9,25]. In this study, 6-OHDA and MPP^+^ were used to induce PD-related phenotypes in PC12 and SH-SY5Y cells. As apoptosis is one of the most important mechanisms of dopaminergic neuron loss in the pathogenesis of PD, inhibiting neuronal apoptosis has been considered a key factor in the search for novel compounds that can protect against PD.

In this study, we found that artemisinin significantly improved the survival rate and decreased apoptosis of PC12 and SH-SY5Y cells incubated with 6-OHDA and MPP^+^. It is generally believed that mitochondria are the main executors of cellular apoptosis [26], and their dysfunction plays a key role in the process of neuronal apoptosis that initiates PD [27]. The decrease of mitochondrial membrane potential is an important marker of the early stage of apoptosis [28]. In this study, we observed that the loss of mitochondrial membrane potential of PC12 and SH-SY5Y cells induced by MPP^+^ or 6-OHDA was significantly prevented by treatment with artemisinin. In addition, we also observed that artemisinin inhibited the increase in LDH release and intracellular ROS accumulation. These results suggest that artemisinin can protect both cell types from oxidative stress induced by 6-OHDA or MPP^+^ and may have potential in the prevention and treatment of PD. Similar results were found in primary cultured mouse neurons. These findings are consistent with our previous studies that have reported the antioxidant activity of artemisinin [10,16,29,30].

In the subsequent in vivo experiments, we also observed the protective effect of artemisinin. The MPTP-induced PD animal model is one currently of the most widely used methods [31], and it is suitable for modeling in mice and non-human primates. In recent years, the neuroprotective effect of artemisinin on the MPTP-induced PD mouse model has also been studied [32]. Particularly, C57/BL6 strain mice are the most sensitive to MPTP [33]. After intraperitoneal injection of MPTP (30 mg/kg) in C57/BL6 mice, the motor coordination ability of mice was significantly decreased. They exhibited symptoms such as tremors, bradykinesia, muscle rigidity, and gait instability. The results of the pole test showed that the turning time and climbing downtime of the MPTP model group were significantly longer than those of the blank control group, indicating slower movement speed and decreased coordination ability. However, the turning time and climbing downtime of the artemisinin group were significantly lower than those of the MPTP-treatment group, and there was a significant improvement in the symptoms of dyskinesia. The open field experiment showed that the number of crossing lines in the model group was significantly lower than that of the control group, while the number of crossing lines in the artemisinin group was significantly higher than that of the PD model group. Additionally, the number of rearing behaviors in the model group was significantly lower than that of the control group, whereas the number of rearing behaviors in the artemisinin group was significantly higher than that in the model group. The same results were just observed in the 6-OHDA induced animal model of PD. Artemisinin administration to the PD animal model resulted in the recovery of behavioral deficits and the attenuation of pathological damage. Therefore, artemisinin may be a promising approach for the treatment of PD.

In recent years, ERK1/2 has been widely recognized as an important signaling pathway involved in the occurrence and progression of PD [34,35]. In this study, the assessment of the mechanisms underlying the protective action of artemisinin suggested the involvement of ERK1/2 signaling in both cellular and animal models. Artemisinin stimulated the phosphorylation of ERK1/2 in a time and dose-dependent manner. It also increased the protein levels of ERK1/2, including P-C-Raf, P-MEK and P-CREB, both upstream and downstream. The ERK1/2 signaling pathway has also been reported to play a key role in mediating oxidative stress-induced apoptosis [36,37,38]. Additionally, the up-regulation of ERK1/2 signaling activity is associated with a neuroprotective effect in both in vivo and in vitro models of PD [38,39]. According to our results, the activation of the ERK1/2 pathway plays a critical role in the neuroprotective effect of artemisinin against MPP+ and 6-OHDA induced injury. Upon inhibiting ERK1/2, the protective effect of artemisinin against cell viability loss, intracellular ROS accumulation, mitochondrial membrane potential decrease, cell apoptosis in vitro, and behavioral deficits and brain pathological damage in vivo were significantly attenuated. In addition, studies have shown that the role of the Transcription Factor EB (TFEB) signaling pathway and the PI3K/Akt signaling pathway in PD is also worthy of attention [40,41]. The TFEB signaling pathway plays a key role in regulating autophagy function, while the PI3K/Akt signaling pathway mediates various biological functions, including inflammation, autophagy, oxidative stress, etc. These signaling pathways are closely related to the ERK1/2 signaling pathway, and there are complex interactions among inflammation, autophagy, and oxidative stress. certainly, we should also take into account the low bioavailability of artemisinin, which may limit its use in disease prevention and may require structural optimization [42]. However, These studies also suggest that the anti-PD effect of artemisinin needs more in-depth study.

## 4. Materials and Methods

### 4.1. Materials

Analytical-grade artemisinin was purchased from Dalian Meilun Biotechnology, Dalian, China. Dulbecco’s Modified Eagle’s Medium (DMEM), Fetal Bovine Serum (FBS), Bovine Serum Albumin (BSA) and Trypsin (0.5% EDTA) were obtained from GIBCOTM (Invitrogen Corporation, New York, NY, USA). A penicillin/streptomycin combination and LipofectamineR 3000 reagent were purchased from Invitrogen Co. (Carlsbad, CA, USA). DMSO was obtained from Sigma Aldrich (St. Louis, MO, USA). Sodium Azide (NaN3) was obtained from Acros Organic (Morris Plains, NJ, USA), and 3-(4,5-dimethylthiazol-2-yl)-2,5-diphenyl tetrazolium bromide (MTT), Hoechst 33,342 were purchased from Molecular Probes (Eugene, OR, USA). DCFH-DA reagent and 5,5′,6,6′-tetrachloro-1,1′,3,3′-tetraethyl-benzimidazolyl-carbocyanineiodide (JC-1) were ordered from Beyotime Institute of Biotechnology, Pierce BCA protein assay kit and HaltTM protease and phosphatase inhibitor cocktail were purchased from Thermo Scientific USA, and Annexin V-FITC/PI was purchased from BBI Life Sciences. Anti-p-ERK1/2, anti-ERK1/2, anti-p-AKT (ser473), anti-C-Raf, anti-p-MEK, anti-p-CREB (ser133), anti-Bax, anti-Bcl2 and anti-GAPDH antibodies were purchased from Cell Signaling Technology (CST) (Beverly, MA, USA). Anti-Rabbit IgG HRP-conjugated secondary antibody was purchased from Promega (Madison, WI, USA). 6-OHDA, MPTP, and MPP+ were purchased from Sigma-Aldrich (St. Louis, MO, USA). PD98059 was purchased from Calbiochem (San Diego, CA, USA).

### 4.2. Cell Culture and Treatments

The cell lines PC12 and SH-SY5Y were obtained from Sun Yat-sen University’s Cell Bank (Guangzhou, China). The cells were grown in DMEM supplemented with 10% FBS supplemented with 10 U/mL penicillin, and 10 μg/mL streptomycin, and maintained at 37 °C in a humidified atmosphere of 5% CO_2_. Primary cultured neurons were prepared from ventral mesencephalon of newborn C57BL/6 mice as previously described [9]. and cultured for 7–8 days in neurobasal medium supplemented with 2% B27, 10 U/mL penicillin, and 10 μg/mL streptomycin at 37 °C in 5% CO_2_ humidified atmosphere.

### 4.3. MTT Assay

MTT assay was used to detect cell viability. The cells were inoculated in 96-well plates at a density of 1 × 10^4^ cells/mL. On the second day, after the cells were completely adherent, the drug was diluted to a suitable concentration using the medium, and then it was used to culture the cells for a specified time. After that, 20 uL MTT (0.5 mg/mL) was added to each well, and the culture was continued for 3 h. Finally, the medium was discarded and DMSO (100 μL/well) was added to dissolve the formazan crystal. The absorbance at 570 nm was measured using Infinite^®^ 200 PRO Nano Quant Multimode Microplate Reader (Tecan Trading AG, Männedorf, Switzerland). Each group had 5 replicates. The detection was repeated at least 3 times.

### 4.4. LDH Assay

Determination of the activity of lactate dehydrogenase (LDH) released into the medium was used to assess cytotoxicity [16]. The cells were inoculated in 96-well plates at a density of 1 × 10^4^ cells/mL. After the cells were treated with drugs, the operation was performed according to the instructions in the kit, and the LDH activity released by the cells into the medium was detected. Finally, the Infinite M200 PRO multi-mode microplate was used to measure the fluorescence intensity. The excitation and emission wavelengths used in the measurement were 560 nm and 590 nm, respectively. LDH values were standardized as the percentage of the control group.

### 4.5. Measurement of Reactive Oxygen Species (ROS)

DCFH-DA Reagent method was used to detect the level of reactive oxygen species (ROS) in cells. The cells were inoculated in a 96-well plate at a density of 1 × 10^4^ cells/mL. The cells were pretreated with artemisinin at a concentration of 6.25 μM for 2 h, and then incubated with or without 6-OHDA for 24 h. Subsequent experimental operations were performed according to the manufacturer’s instructions. The cells were cultured in a fresh medium containing DCFH-DA Reagent and incubated at 37 °C for 1 h. After reaching the specified time, the cells were gently washed twice with PBS. Finally, the image was taken with a fluorescence microscope.

### 4.6. JC-1 Staining

Mitochondrial membrane potential was measured by JC-1 staining. The cells were seeded in 96-well plates at a density of 1 × 10^4^ cells/mL. The cells were pretreated with 6.25 μM artemisinin for 2 h, and then incubated with or without MPP^+^ or 6-OHDA for 24 h. Following the manufacturer’ s instructions, cells were incubated with JC-1 dye (10 mg/L) in a fresh medium. The temperature was 37 °C and the incubation time was 30 min. After the treatment was completed, the cells were washed twice with PBS. Then. The intensities of red fluorescence (excitation 585 nm, emission 590 nm) and green fluorescence (excitation 514 nm, emission 529 nm) were captured by an infinite M200 PRO multimode microplate reader fluorescence microscope. Mitochondrial membrane potential (Δψm) was measured by the ratio of red/green fluorescence intensity. And normalized with the control group. The mitochondrial membrane potential (Δψm) was measured by the ratio of red/green fluorescence intensity, and then normalized with the control group.

### 4.7. Flow Cytometry

The cells were seeded in 96-well plates at a density of 1 × 10^4^ cells/mL. The cells were pretreated with 6.25 μM artemisinin for 2 h, and then incubated with or without MPP^+^ or 6-OHDA for 24 h. After incubation, cells were collected and washed twice with cold PBS. After cell counting, the cells were resuspended with buffer at a density of 1 × 10^6^ cells/mL and then loaded into a flow tube. The cells were stained with Annexin V-FITC (10 μL) and propidium iodide (PI) (5 μL) in the dark for 15 min. Flow cytometry was performed using FACS instruments (BD Accuri C6, Franklin Lakes, NJ, USA).

### 4.8. Western Blot Analysis of Cell Samples

In accordance with previous descriptions, Western blotting was performed [11]. Briefly, the samples were placed on ice and lysed after adding a protein lysis buffer containing protease inhibitors and phosphatase inhibitors. The protein concentration was determined using the BCA protein concentration determination kit. After unifying the protein concentration and the sample volume, SDS-PAGE was used for separation, and then transferred to the PVDF membrane. After blocking the membrane with 5% skim milk, the protein was detected using the appropriate antibody (Table 1). Image J software 16-bit Unsigned was used to quantify the optical density of the band intensity.

### 4.9. CRISPR/Cas9 Knockout of ERK1/2 Gene

The sequence of MAPK1(ERK2)-sgRNA-oligo1 was CACCGTGGCAGAGATGCTATCCAAC, oligo2 was AAACGTTGGATAGCATCTCTGCCAC; and the sequence of MAPK3(ERK1)-sgRNA oligo1 was CACCGCCACTCTGGTCTTGCGCACG, oligo2 was AAACCGTGCGCAAGACCAGAGTGGC. Briefly, PC12 cells were cultured in 12-well plates at the density of 3 × 10^5^ cells/well for 24 h. The plasmids expressing Cas9 with ERK1-sgRNA, ERK2-sgRNA and puromycin resistant gene were co-transfected into PC12 cells using Lipofectamine 3000 reagent. After 48 h, cells were added puromycin at a concentration of 2 μg/mL, and clones are selected and harvested for immunoblotting analysis with ERK1/2 antibody or other assays.

### 4.10. Hoechst 33342 Staining

Hoechst 33342 staining was used to detect apoptosis. The cells were seeded in a 96-well plate at a density of 1 × 10^4^ cells/mL, and pretreated with 50 μM PD98059 (ERK inhibitor) for 60 min, followed by pretreatment with artemisinin at a concentration of 6.25 μM for 2 h, and finally incubated with or without MPP^+^ or 6-OHDA for 24 h. After incubation, the cells were incubated with Hoechst 33342 dye in a fresh medium at room temperature for 5 min. Then, the cells were washed twice with PBS. Images were collected using a fluorescence microscope (EVOS FL imaging system).

### 4.11. Mouse Models of Parkinson’s Disease

#### 4.11.1. Animals

We obtained male C57BL/6 mice (8-week-old) from the Animal Research Core and placed them in the University of Macau’s Animal Facility. Animal experiments were conducted in accordance with guidelines approved by the University of Macau Animal Ethics Committee (protocol no.: UMAEC-13-2015). Water and food were readily available, and the animal housing conditions were regulated (temperature 23 °C, humidity 60–65%, and light from 7:00 to 19:00).

#### 4.11.2. Establishment and Grouping of the 6-OHDA-Induced Parkinson’s Disease Model

8 μg 6-OHDA (Sigma-Aldrich, St. Louis, MO, USA) dissolved in 2 μL 0.2% saline with ascorbic acid were administered in a stereotactic surgery into the right striatum at the following coordinates: anterior/posterior: −0.9 mm; medial/lateral: +1.8 mm; ventral/dorsal: −3.0 mm. After the infusion, the syringe was maintained in the brain for an additional 5 min before it was slowly retracted [43,44,45]. Controls were injected with an equal volume (2 μL) of the vehicle consisting of 0.2% saline with ascorbic acid. After the procedure, the animals were returned to their cages for recovery. The animals were randomly divided into a saline control group, 6-OHDA model group, ART treatment group (5 mg/kg), and ART plus PD98059 treatment group, with fifteen mice per group. ART was dissolved in 2% DMSO in physiological saline and was given via intraperitoneal (i.p.) injection. PD98059 (a special inhibitor of ERK) was dissolved in 2% DMSO in physiological saline and was administered via i.p. injection. The animals from the control and model groups were i.p. injected with equal amounts of normal saline. The effects of ART on 6-OHDA-induced parkinsonism were studied in the following experimental groups: Control group, saline (icv.) + saline (i.p.); Model group, 6-OHDA (icv.) + saline (i.p.); ART group, 6-OHDA (icv.) + ART (5 mg/kg/day, i.p.); PD98059 group, 6-OHDA (icv.) + ART (5 mg/kg/day, i.p.) + PD98059 (10 mg/kg/day, i.p.). The animals were treated daily for 7 days. After the last administration and behavioral assessment, the mice were euthanized and the brain was removed and fixed in 4% paraformaldehyde overnight, then embedded in paraffin for both histological and immunofluorescence examinations or stored at −80 °C for Western blot analyses.

#### 4.11.3. Establishment and Grouping of the MPTP-Induced Parkinson’s Disease Model

Mice were randomly divided into a control group, MPTP (30 mg/kg/d) model group, and ART + MPTP group (ART 5 mg/kg/d, MPTP 30 mg/kg/d), with ten mice per group. In the ART + MPTP group, ART was dissolved in 2% DMSO in physiological saline and given 1 h before MPTP treatment. In the MPTP model group, an equal amount of DMSO (2%) in normal saline as a control of ART treatment was given 1 h before that MPTP (Sigma, St. Louis, MO, USA) dissolved in physiological saline was given to induce Parkinson’s disease. In the control group, the mice were given an equal amount of DMSO (2%) in normal saline as a control of ART treatment 1 h prior to an equal amount of normal saline as a control of MPTP treatment. All the treatment was given via i.p. daily for 7 consecutive days and the behavior was assessed on the 8th day.

### 4.12. Neurobehavioral Observation Method

#### 4.12.1. Open Field Test

Mice’s locomotor activity can be quantified by an open field experiment consisting of spontaneous exercise. The assay used a video analysis system to capture activity in the open field. Observations were conducted continuously for five minutes within observation boxes (length 50 cm, width 50 cm, height 50 cm). Once the recording was complete, the system performed an automatic trajectory analysis. The 5-min translation distance and the number of central entries were obtained as a motion indicator. The environment was kept absolutely quiet during the experiment. In order to remove any interference from odors, the observation boxes were also wiped clean with alcohol on a cotton ball.

#### 4.12.2. Pole Test

The pole test is a useful method to measure motor coordination and balance in several mice models of PD [46]. Briefly, On top of a 60 cm high and 1 cm diameter rough-surfaced pole, mice were placed head downwards. It was recorded how long it took to climb down the pole. For each mouse, three trials were performed, and their motor function was assessed based on the average value of the trials.

#### 4.12.3. Swimming Test

The swimming test is a reliable method of detecting mice’s coordination of limb movement. We placed mice in a circular pool with a diameter of 120 cm and a height of 40 cm. A software program was used to track and record a mouse’s movement trajectory within one minute, and to analyze the total swimming distance and average speed of mice within that time.

#### 4.12.4. Rotarod Test

During a five-min experiment, mice were placed on a shifting rod whose rotation speed increased from 0 rpm to 40 rpm. We recorded how long mice stayed on the rod, their drop speed, and their total distance traveled. A minimum of three trials spaced at least 1 h apart were used to calculate the average.

### 4.13. H&E, Nissl Staining, and Immunofluorescence

In accordance with previously described protocols, H&E and Nissl stainings were performed [47]. H&E staining was used to observe the morphology of cells and Nissl staining was used to detect motor neurons. The survival index was calculated as follows: survival index (%)  =  (number of motor neurons/total number of neurons) × 100% Immunofluorescence analyses were performed as previously described [48]. Briefly, slides were prepared. After incubation with antigen retrieval solution for 30 min, the slides were rinsed with PBS and incubated with the primary antibodies (rabbit anti-TH, 1:500, mouse anti-GFAP, 1:500, rabbit anti-P-ERK, 1:500) overnight at 4 °C. On the next day, the slides were rinsed and incubated with the corresponding secondary antibody (FITC-conjugated anti-mouse IgG, FITC-conjugated anti-rabbit IgG) for 2 h followed by washing in PBS for 15 min. The images were acquired with a fluorescence microscope (Carl Zeiss LSM710 Confocal, Carl Zeiss, Jena, Germany).

### 4.14. Western Blot Analysis of Brain Tissue

To 10 mg of minced tissue, we added 1 mL of RIPA lysis buffer and 10 L of 100 mM PMSF, and homogenized it. After centrifugation at 14,000 rpm for 5 min at 4 °C, the supernatant was collected. Assaying protein concentrations with a BCA protein assay kit and separating equal amounts of proteins by SDS-PAGE, followed by PVDF membrane transfer, were performed. Proteins were probed with antibodies after blocking the membrane with 5% skim milk. With the help of Image J, the intensity of the bands was quantified.

### 4.15. Statistical Analysis

Each condition was tested in triplicate in one well. In vitro experiments were conducted in triplicate. A minimum of three wells were used for each condition in in vivo experiments. GraphPad Prism 8.0 statistical software (GraphPad software, Inc., San Diego, CA, USA) was used to assess data significance. All values were presented as mean ± SD. Statistical significance among various groups was calculated by one-way ANOVA using post hoc multiple comparisons, when *p* < 0.05 was considered statistically significant.

## 5. Conclusions

**Figure 13:**
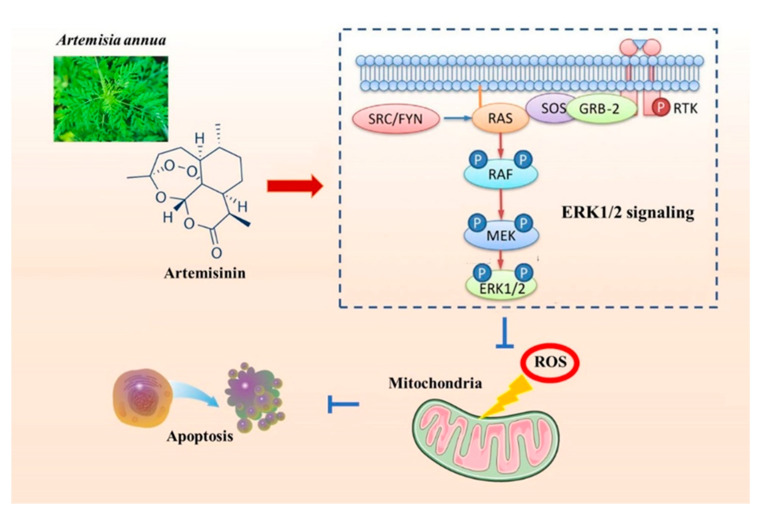
Putative mechanism of artemisinin neuroprotective action against 6-OHDA and MPP^+^-induced neuronal injury. Artemisinin stimulated ERK1/2 related signaling pathway in neuronal cells and the brain of the PD mice model, resulting in the reduction of oxidative stress, correction of mitochondrial dysfunction, and inhibition of apoptosis.

In conclusion, our study offers evidence that artemisinin has a protective effect against 6-OHDA and MPP+ induced injury in vitro and in vivo by ERK1/2 activation (Figure 13). Our findings support the potential use of artemisinin in the development of more effective therapeutic approaches against PD.

## Figures and Tables

**Table 1:** Antibody Information.

Antibody	Cat.NO	Source	Dilution Ratio	Company
Phospho-Erk1/2 (Thr202/Tyr204)	9101S	Rabbit	1:1000	CST
ERK 1/2 Polyclonal	40902	Rabbit	1:1000	SAB
P-AKT (S473)	4060	Rabbit	1:1000	CST
Phospho-c-Raf (Ser259)	9421	Rabbit	1:1000	CST
Phospho-MEK1/2 (Ser217/221) (41G9)	9154	Rabbit	1:1000	CST
Phospho-CREB (Ser133) (87G3)	9198	Rabbit	1:1000	CST
Bax	34260-2	Rabbit	1:1000	SAB
Bcl-2	32012	Rabbit	1:1000	SAB
Anti-rabbit IgG, HRP-linked Antibody	7074		1:5000	CST

## Data Availability

The study did not report any data.
